# Development and Evaluation of Maze-Like Puzzle Games to Assess Cognitive and Motor Function in Aging and Neurodegenerative Diseases

**DOI:** 10.3389/fnagi.2020.00087

**Published:** 2020-04-21

**Authors:** Tobias Nef, Alvin Chesham, Narayan Schütz, Angela Amira Botros, Tim Vanbellingen, Jean-Marc Burgunder, Julia Müllner, René Martin Müri, Prabitha Urwyler

**Affiliations:** ^1^Gerontechnology & Rehabilitation, University of Bern, Bern, Switzerland; ^2^ARTORG Center for Biomedical Engineering Research, University of Bern, Bern, Switzerland; ^3^Neurocenter, Luzerner Kantonsspital, Lucerne, Switzerland; ^4^Neurozentrum Siloah and Department of Neurology, Swiss Huntington’s Disease Center, University of Bern, Bern, Switzerland; ^5^Department of Neurology, University Neurorehabilitation, Inselspital, Bern, Switzerland

**Keywords:** cognitive assessment, maze task, puzzle video games, aging, neurodegenerative diseases

## Abstract

There is currently a need for engaging, user-friendly, and repeatable tasks for assessment of cognitive and motor function in aging and neurodegenerative diseases. This study evaluated the feasibility of a maze-like Numberlink puzzle game in assessing differences in game-based measures of cognition and motor function due to age and neurodegenerative diseases. Fifty-five participants, including young (18–31 years, *n* = 18), older (64–79 years, *n* = 14), and oldest adults (86–98 years, *n* = 14), and patients with Parkinson’s (59–76 years, *n* = 4) and Huntington’s disease (HD; 35–66 years, *n* = 5) played different difficulty levels of the Numberlink puzzle game and completed usability questionnaires and tests for psychomotor, attentional, visuospatial, and constructional and executive function. Analyses of Numberlink game-based cognitive (solving time and errors) and motor [mean velocity and movement direction changes (MDC)] performance metrics revealed statistically significant differences between age groups and between patients with HD and older adults. However, patients with Parkinson’s disease (PD) did not differ from older adults. Correlational analyses showed significant associations between game-based performance and movement metrics and performance on neuropsychological tests for psychomotor, attentional, visuospatial, and constructional and executive function. Furthermore, varying characteristics of the Numberlink puzzle game succeeded in creating graded difficulty levels. Findings from this study support recent suggestions that data from a maze-like puzzle game provide potential “digital biomarkers” to assess changes in psychomotor, visuoconstructional, and executive function related to aging and neurodegeneration. In particular, game-based movement measures from the maze-like puzzle Numberlink games are promising as a tool to monitor the progression of motor impairment in neurodegenerative diseases. Further studies are needed to more comprehensively establish the cognitive validity and test–retest reliability of using Numberlink puzzles as a valid cognitive assessment tool.

## Introduction

Neurodegenerative diseases comprise a heterogeneous group of age-related disorders with progressive loss of neuronal structure and function (Cummings and Pillai, 2016; Kovacs, 2018). Neurodegeneration can occur in normal aging and age-related neurodegenerative diseases that include Alzheimer’s disease (AD), Parkinson’s disease (PD), and Huntington’s disease (HD). Affecting different brain areas, patients with neurodegenerative diseases suffer from cognitive, motor, and neuropsychiatric impairments (Cummings and Pillai, 2016; Hussain et al., 2018; Kovacs, 2018). Cognitive functions commonly affected in neurodegenerative diseases include attentional, visuospatial, and constructional, executive, and motor function that detrimentally impact everyday activities and quality of life in normal cognitive aging (Deary et al., 2009; Diesing and Rizzo, 2018), AD (Rycroft and Giovannetti, 2017), PD (Ding et al., 2015; Pal et al., 2018), and HD (Dumas et al., 2013; Paulsen and Long, 2014). Strikingly, these cognitive changes vary considerably both between and within individuals in normal aging (Agrigoroaei and Lachman, 2011; Mosti et al., 2019) and patients with neurodegenerative disorders (Boller et al., 2013; White et al., 2018; Greenland et al., 2019).

Given this variability, it is important to differentiate normal cognitive aging from neurodegenerative diseases in order to detect cognitive deficits early and provide adequate treatment to attenuate further neurodegeneration and cognitive dysfunction (Massaldjieva, 2018). However, existing neuropsychological assessments have been criticized for their: (a) unsuitability for repeated use because of time demand and practice effects (i.e., test performance improvements when tests are taken repeatedly); (b) limited sensitivity to detect early and subtle cognitive changes because of ceiling and flooring effects (i.e., high rates of highest or lowest possible test scores); and (c) lack of ecological validity as many cognitive tasks do not reflect cognitive demands of everyday activities (Silverberg et al., 2011; Allard et al., 2014; Zygouris and Tsolaki, 2015; Valdes et al., 2016; Howieson, 2019).

A novel approach to address these issues is the use of video games to study age and neurodegenerative disease-related differences in cognitive function (Boot, 2015; Koo and Vizer, 2019). Video games involve solving cognitive challenges that require a range of cognitive abilities and often share properties with psychological tests (Holmgard et al., 2016). Video games provide a bulk of performance measures (e.g., reaction and solving time, number of errors and others) that can be used to assess cognitive functioning (Areàn et al., 2016; Mandryk and Birk, 2019). Furthermore, video games promote prolonged motivation and engagement, and offer performance-based adjustments of task difficulty that can help avoid practice effects with repeated use and accommodate different levels of cognitive ability (Holmgard et al., 2016; Diesing and Rizzo, 2018; Levy et al., 2018). Also, video games involve complex cognitive skills that better reflect everyday cognitive function than simple cognitive tasks (Boot et al., 2013). To date, several studies demonstrated associations between video game performance measures and performance on specific cognitive tests, suggesting that video games measure relevant cognitive abilities (Baniqued et al., 2013; Oei and Patterson, 2013; Martinovic et al., 2015) and are feasible to assess cognitive function in older adults (Thompson et al., 2012; Tong et al., 2016).

With regard to detecting age-related changes in cognitive and motor functioning, maze tasks represent a particularly promising addition to standardized cognitive assessments (de Souza et al., 2013). Mazes are complex visual-motor planning and problem-solving tasks that require finding a path from the start to the end of a maze as quickly as possible. Mazes are non-verbal, simple to understand and use, relatively independent of educational level and suitable for a wide range of older adults and persons with cognitive impairment (Marhasev et al., 2009; Silva et al., 2017). Maze tasks require an interplay between both cognitive and motor processes and are similar to complex everyday activities that require planning and problem solving (Lewis and Miller, 2007; Marhasev et al., 2009; Howieson, 2019). Solving mazes requires multiple cognitive processes that include attentional, visuospatial and visuoconstructional, and executive function (planning, foresight and problem solving) as well as visuomotor function (Snellgrove, 2005; Kirsch et al., 2006; Carlozzi, 2011; Zhao and Marquez, 2013).

To date, maze tests have been used in some studies to assess cognitive and motor functioning and demonstrated sensitivity in differentiating healthy normal aging from either mild cognitive impairment (Zhang et al., 2007; de Souza et al., 2013), Alzheimer’s dementia (Mack and Patterson, 1995), PD (Mimura et al., 2006), and HD (Deckel and Duffy, 2000; Montoya et al., 2006). Furthermore, maze task performance was shown as a strong predictor of everyday functioning ability in older adults (Mack and Patterson, 1995; Ott et al., 2003; Lewis and Miller, 2007; Staplin et al., 2013).

In the current study, a maze-like Numberlink (NL) puzzle video game adapted to assess cognitive and motor ability in aging and neurodegenerative diseases is presented. NL was first published as a newspaper puzzle column by Sam Loyd (Loyd, 1897) and later popularized in Japan as pastime puzzle books (Dudeney, 1917; Nikoli, 1989; Yoshinaka et al., 2012; Adcock et al., 2015). In 2012, NL puzzles have been released as a puzzle game for mobile applications called Flow Free^®^ (Big Duck Games LLC) that has been downloaded over 250 million times and is among the most popular puzzle games (Newman and Newman, 2012). In a previous playtest study in older adults, we found that the NL puzzles were particularly enjoyed and rated to meet game characteristics for cognitive tasks and trainings (Chesham et al., 2017).

Similar to video games, mazes can be modified in difficulty to match the test-takers’ level of cognitive ability. A number of recent studies have further provided maze tasks with graded levels of difficulty accomplished by varying variables of the maze (McClendon, 2001; Ott et al., 2003; Blatter et al., 2005; Davis et al., 2014; Pasek, 2016). Variable maze difficulty levels can help prevent practice effects during repeated administration and reduce ceiling and flooring effects by continuously matching the task difficulty to the participant’s cognitive ability level (Davis et al., 2014; Loe and Rust, 2019). Graded difficulty levels for the NL puzzle game were generated by manipulating game characteristics. This benefits future studies to create game-based adaptive computerized cognitive assessments.

The first aim of this study was to examine the acceptance and usability of a game-based NL task in young, older, and oldest adults and persons with neurodegenerative diseases. In line with recent suggestions, we hypothesize that the use of playful elements of a puzzle video game is user-friendly and enjoyable for older people and patients with neurodegenerative diseases (Polzer and Gewald, 2019). The second aim of this study was to examine age- and NDD related differences in NL game-based cognitive (solving time and accuracy) and motor (movement velocity and direction changes) performance.

Following previous findings using maze tasks, our study tests the hypothesis that NL game-based performance measures can be used to distinguish between age groups (de Souza et al., 2013) and between healthy controls and patients with HD (Montoya et al., 2006) and PD (Mimura et al., 2006). Based on our hypothesis that NL puzzles are similar to maze tasks, we propose that NL game-based performance measures are associated with performance on the Snellgrove Maze Task (SMT) and standard measures of visuomotor, visuospatial and constructional, executive, and global cognitive function (Snellgrove, 2005; Yew et al., 2011). Finally, we assessed whether NL puzzle difficulty can be varied by manipulating game parameters. Following a previous study, we hypothesize that the difficulty of NL puzzles increases with set size and the number of paths (van Kreveld et al., 2015).

## Materials and Methods

### Participants

In total, 55 participants (36 females) between the ages of 18 and 98 years participated in this study (see Table 1). Five groups of participants were enrolled for this study: (1) young adult university students (YA; *n* = 18, 12 female, mean age 21.83, range 18–31 years); (2) older adults (OA; *n* = 14, 8 female, mean age 71.14, range 64–79 years); (3) oldest adults (OOA; *n* = 14, 12 female; mean age 89.93, range 86–98 years) living independently in senior residence apartments. The group of participants with neurodegenerative disease included; (4) patients with HD (*n* = 5, 2 female; mean age 49.40, range 35–66 years; Movement Disorder Society – Unified Parkinson’s Disease Rating Scale (MDS-UPDRS-III) 45.75 ± 10.78, range 30–54; Total Functional Capacity 8.0 ± 3.16, range 6–12; Functional Assessment: 15.75 ± 6.18, range 7–21; Independence Scale 81.25 ± 21.75%, range 60%–100%); and (5) patients with PD (*n* = 4, 2 female; mean age 67.50, range 59–76 years; MDS-UPDRS-III 17.75 ± 5.38, range 11.00–23.00; disease duration 9.88 ± 3.01 years, range 7.00–14.00 years). HD and PD patients were recruited from the Swiss HD Center, Neurozentrum Siloah; Department of Neurology, Bern, Switzerland; and the Neurology and Neurorehabilitation Center, Luzerner Kantonsspital, Luzern, Switzerland. All HD patients were previously given a clinical confirmed diagnosis of HD. One of five HD patients did not receive any medication, while the rest of HD patients were under medications. The medications were personalized depending on the individual symptoms and covered a wide range (Zolpidem 10 mg 1–2, Risperidone 0.5 mg, Trittico 100 mg, Citalopram 20 mg, Quetiapine 25 mg, Zoldorm 10 mg, Aripiprazole 5 mg, Eltroxin LF 0.05 mg, and Rosuvastatin 10 mg). Patients with PD were diagnosed with PD as per UK Parkinson’s Disease Brain Bank criteria and prescribed anti-Parkinsonian medication (levodopa-dose 412.5 ± 188.75 mg, range 150–600 mg). Three of the PD patients were also prescribed additional drugs depending on their symptoms (Sifrol ER 1.5 mg, Pramipexole 3 mg, Roprinirole 8 mg, and Safinamide 50 mg). All PD patients were in “ON” phase of the medication cycle when participating in this study.

**Table 1:** Demographics, clinical and neuropsychological measures, acceptance, and usability by group.

	YA (*n* = 18)	OA (*n* = 14)	OOA (*n* = 14)	PD (*n* = 4)	HD (*n* = 5)	Statistics
	*Demographic variables*				
Age (years)	21.83 ± 3.28	71.14 ± 4.31	89.93 ± 3.50	67.50 ± 6.95	49.40 ± 13.32	χ^2^ = 568.21^Z^, *p* < 0.001^Z,A,B,I^, 0.023^D^, 1.000^C,F,G,J^, 0.011^E^, 0.001^H^, 0.038^I^
Sex [m:f (%f)]	6:12 (66.7)	6:8 (57.1)	2:12 (85.7)	2:2 (50.0)	3:2 (40.0)	χ^2^ = 8.16^Z^, *p* = 0.305^Z^
	*Neuropsychological measures*				
TMT-A (s)	26.44 ± 9.38	36.15 ± 7.69	66.89 ± 33.07	45.25 ± 11.50	98.20 ± 79.95	χ^2^ = 29.43^Z^, *p* < 0.001^Z,B^, 1.000^G,H,I,J^, 0.047^A^, 0.021^D^, 0.001^C^, 0.002^E^, 0.455^F^
TMT-B (s)	50.78 ± 19.30	107.69 ± 43.21	145.50 ± 18.72	122.50 ± 68.73	227.00 ± 17.89	χ^2^ = 32.13^Z^, *p* < 0.001^Z,B,C^, 0.001^A^, 1.000^E,G,H,I^, 0.019^D^, 0.013^F^, 0.074^J^
SMT (s)	14.39 ± 3.13	32.23 ± 10.12	43.44 ± 20.34	24.75 ± 11.12	50.40 ± 35.19	χ^2^ = 32.56^Z^, *p* < 0.001^Z,A,B,C^, 0.009^I^, 1.000^D,E,F,G,H,J^
MoCA score	28.61 ± 1.54	27.23 ± 2.86	23.44 ± 4.19	26.25 ± 3.40	18.30 ± 6.2	χ^2^ = 19.18^Z^, *p* < 0.001^Z,B,C^, 1.000^A,D,G,H,I,J^, 0.005^E^, 0.008^F^
UPDRS III^Q^				17.75 ± 5.38	45.75 ± 10.78	F = 21.60^J^, *p* = 0.004^J^
	*Perception of game training questionnaire ratings and usability*				
Enjoyable^P^	6.28 ± 0.75	6.14 ± 1.56	6.64 ± 0.50	6.50 ± 0.58	5.80 ± 1.10	χ^2^ = 3.81^Z^, *p* = 0.432^Z^
Challenging^P^	2.78 ± 1.56	3.86 ± 1.61	2.86 ± 1.92	5.00 ± 1.41	4.20 ± 2.17	χ^2^ = 8.75^Z^, *p* = 0.068^Z^
Frustrating^P^	1.00 ± 0.00	1.29 ± 0.83	1.29 ± 0.47	1.50 ± 0.58	1.60 ± 0.89	χ^2^ = 8.98^Z^, *p* = 0.062^Z.^
Motivating^P^	6.56 ± 0.62	6.64 ± 0.50	6.64 ± 0.63	7.00 ± 0.00	6.60 ± 0.55	χ^2^ = 2.36^Z^, *p* = 0.669^Z^
SUS score	93.38 ± 5.72	93.33 ± 6.25	83.39 ± 11.79	81.88 ± 9.66	83.12 ± 10.68	χ^2^ = 12.04^Z^, *p* = 0.017^Z^, 1.000^A,H,I,J^, 0.140^B^, 0.609^C^, 0.355^D^, 0.228^E^, 0.689^F^, 0.414^G^.

*All values, mean ± SD or *n* (%), *p* value < 0.05 is significant. YA, Young Adults; OA, Older Adults; OOA, Old oldest Adults; PD, Parkinson’s Disease; HD, Huntington’s disease; TMT, Trail Making Test; SMT, Snellgrove Maze Task; MoCA, Montreal Cognitive Assessment (Score: 1–30), ^P^Perception of Game Training Questionnaire (Rating: 1–7); SUS, System Usability Scale (Score: 0–100), ^Q^Unified Parkinson’s Disease Rating Scale, ^Z^comparison across all groups df = 4, ^A^YA vs. OA, ^B^YA vs. OOA, ^C^YA vs. PD, ^D^YA vs. HD, ^E^OA vs. OOA, ^F^OA vs. PD, ^G^OA vs. HD, ^H^OOA vs. PD, ^I^OOA vs. HD, ^J^PD vs. HD*.

Exclusion criteria for participation were insufficient coordinative, motor, and perceptual ability to handle a tablet-computer and history of any additional neurological or psychiatric deficits. All participants had normal or corrected-to-normal vision. Written informed consent was provided in accordance with the latest version of the Declaration of Helsinki prior to participation. The cantonal ethics committees of Bern, Northwest and Central Switzerland, Switzerland granted the ethical approval for this study (2016-01281).

### Neuropsychological Assessment

Global cognitive, attentional, visuospatial and visuoconstructional, and executive function was evaluated in all participants. Global cognitive ability was examined with the Montreal Cognitive Assessment (MoCA) that evaluates executive, attentional and visuospatial function, memory, and language (Nasreddine et al., 2005). Patients with HD completed the Mini Mental State Examination (MMSE; Folstein et al., 1975) instead of the MoCA. MMSE scores for the HD patients were converted to MoCA scores using conversion guidelines from Roalf et al. (2013). Attention and executive functions were further assessed with the Trail Making Test part A (TMT-A) and part B (TMT-B; Schretlen et al., 1996). The TMT-A tests selective attention, visual scanning, and visuomotor processing, and the TMT-B tests divided attention and executive control (Schretlen et al., 1996; Strauss et al., 2006). Finally, the SMT was used as a screening for multiple cognitive functions that include attention, visuoconstructional ability, and executive functions of planning and foresight (Snellgrove, 2005).

### Acceptance and Usability Assessment

Subjective acceptance of the NL puzzle game was assessed with the Perception of Game Training Questionnaire (Boot et al., 2013). In this questionnaire, participants rated the extent to which they found playing the mazes “enjoyable,” “challenging,” “frustrating,” as well as their motivation while playing the mazes on a seven-point Likert scale. The 10-item System Usability Scale (SUS) was used to measure user experience, usability, and learnability of the NL puzzle game. The SUS provides a composite score from 0 to 100 where a higher number indicates a higher usability (Brooke, 1996).

### NL Puzzle Task

#### Task Description

**Figure 1:**
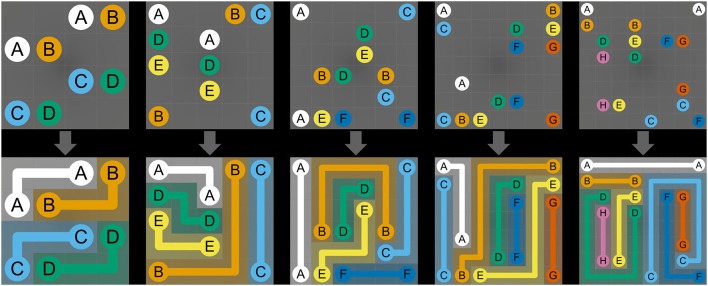
Numberlink puzzle difficulty levels. The manipulated variables include the width and height of the board, the number of paths, and the total number of turns. The top row depicts the initial, and the bottom row, the solved NL puzzles. The difficulty levels shown from left to right are (*width, height, paths, turns*) = (4, 4, 4, 4), (5, 5, 5, 5), (6, 6, 6, 6), (7, 7, 7, 7), (8, 8, 8, 8).

NL puzzles are maze-like link puzzles that involve finding multiple distinct paths to connect pairs of identical objects using non-intersecting and continuous lines (Yew et al., 2012). NL puzzles are played on a grid-based (width × height) puzzle board of cells. Some cells contain colored circles with numbers or letters (“dots”) that represent start and end points of paths, while the rest of the cells are empty (Kalvelagen, 2017). Dots always come in pairs that have the same color and letter (Hartmann, 2018; see Figure 1, top row). The overall goal of NL puzzles is to connect all pairs of dots with non-intersecting and continuous lines (called “path” or “flow”) such that finally each empty cell in the grid is part of a path (Adcock et al., 2015). The following rules must be observed to solve NL puzzles: first, all pairs of identical dots must be connected with single continuous paths. Second, paths can be drawn only in horizontal or vertical direction, must go at least through one empty cell, and cannot go through an empty cell twice. Third, paths are not allowed to cross cells containing dots or intersect with other paths, as crossed paths will be overwritten. Fourth and final, all empty cells must be filled with paths once all pairs of dots are connected (see Figure 1, bottom row; Newman and Newman, 2012; Yew et al., 2012; Yoshinaka et al., 2012; Kalvelagen, 2017; Hartmann, 2018; Laurentiz, 2018). The main challenge of NL puzzles lies in completing the puzzle in as little time as possible using a minimum number of moves. Moves are counted from the moment a dot is tapped and dragged to draw a path until it is released again. Moves can result in either complete or incomplete paths between two identical dots. Paths can be deleted completely by touching the start or end dot or can be broken up at the point where the path is touched. Hence, to solve a NL puzzle with an optimal number of moves, each pair of dots should be connected exactly once; that is, the number of moves should be equal to the number of pairs of dots (Newman and Newman, 2012; van Kreveld et al., 2015).

#### Comparison Between Maze Tasks and NL Puzzles

**Figure 2:**
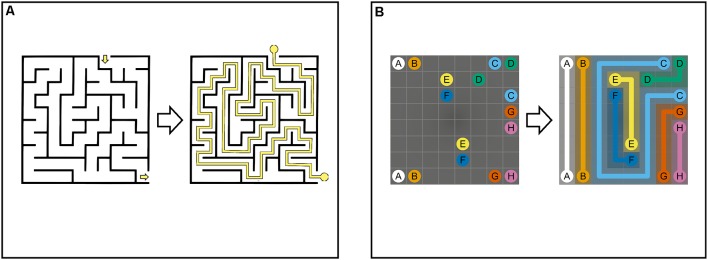
Maze Task **(A)**: maze with cells separated by boundaries. The maze comprises connected paths that are open or closed (dead-ends). To solve the maze, single continuous path (i.e., sequence of connected paths) between start and end must be found. Numberlink Puzzle **(B)**: Numberlink puzzle consisting of a two-dimensional grid with multiple start and end points (i.e., pairs of identical dots). To solve the puzzle, all pairs of dots must be connected with continuous non-intersecting lines and all empty cells must be part of a path.

NL puzzle games share similarities with perceptual maze tasks such as paper-based and computerized versions of the Porteus Maze Tests and Wechsler Mazes (Porteus, 1945; Wechsler, 1949; Ott et al., 2003; Blatter et al., 2005), the Elithorn Perceptual Maze Test (Elithorn, 1955; Loe and Rust, 2019), and the SMT (Snellgrove, 2005). Maze tasks are composed of two-dimensional grids of cells and are made up of a complex set of branching paths (“arms”) confined by fixed walls between cells. The start (“entry”) and end (“exit”) points are located on the outer edge of the maze (Figure 2, Maze Task, left). Arms inside the maze are connected by intersections and are either open or closed (“dead-ends”). The goal of maze tasks is to draw a single continuous path from entry to the exit (Figure 2, Maze Task, right). This requires a multistep solution composed of a set of connected, directed arms within the maze. Therefore, solving a maze involves selecting multiple, consecutive correct arms and avoiding dead-ends at every intersection of the maze (Bagnall and Zatuchna, 2005; Blatter et al., 2005; Carlozzi, 2011; Pasek, 2016).

Like perceptual mazes, NL puzzles are played on a two-dimensional grid of cells. Instead of finding a single path between a single entry and exit point, NL puzzles involve connecting multiple unordered start and end points (i.e., pairs of identical dots) with multiple continuous, non-intersecting paths (Figure 2, NL Puzzle). In addition, unlike mazes, paths in NL puzzles are not delimited by static boundaries, but “pre-determined” by constraints given by the rules of the game (Laurentiz, 2018). For this reason, NL puzzles are considered a set of undirected paths that connect multiple start and end points (Yew et al., 2011; Alviano et al., 2013; Hartmann, 2018).

#### NL Difficulty Level Generation

The NL puzzle game used in this study was adapted according to the Flow Free^®^ App (Big Duck Games LLC; Newman and Newman, 2012). In the Flow Free^®^ App, difficulty is governed by the size of the grid-based board with sizes ranging from 5 × 5 to 15 × 15. A recent study showed that four game characteristics can be used to model and control the difficulty level of NL puzzles: (1) the size of the board (width × height); (2) the number of paths or pairs of dots; (3) the total number of turns, that is, direction changes of paths to the left or right; and (4) the average city block distance between start and end points of paths (Mutser, 2014; van Kreveld et al., 2015).

To create fine-grained and controlled difficulty levels for the NL puzzle in this study, a set of 25 board sizes was specified by all combinations of board widths and heights varied over a range from 4 to 8: (*width, height*) = (4, 5, 6, 7, 8) × (4, 5, 6, 7, 8). For each of the 25 board sizes, 1 Mio. random NL puzzles were computer-generated using an open-source Numberlink Generator (Ahle, 2017). Following a recent study on determinants of NL puzzle difficulty, the generated puzzles (text files) were computer-processed and sorted by the number of paths and the total number of turns (Mutser, 2014; van Kreveld et al., 2015). In order to reduce the number of generated NL puzzles for this study, the number of paths and the number of total turns per maze were set between 4 and the maximum value of the width or height of the board. This resulted in a total number of 361 difficulty levels: (*width, height, paths, turns*) = (4, 5, 6, 7, 8) × (4, 5, 6, 7, 8) × [4 ≤ paths ≤ max (width, height)] × [4 ≤ turns ≤ max(width, height)]. Then, two parallel versions of difficulty levels (see Supplementary Material, Appendix) were created, by parallelizing board size and number of paths (*width, height, paths*). Both sets contained all 15 *square* (*width = height*) NL difficulty levels (*width, height, paths*) = (4, 4, 4), (5, 5, 4), (5, 5, 5), (6, 6, 4), (6, 6, 5), (6, 6, 6), (7, 7, 4), (7, 7, 5), (7, 7, 6), (7, 7, 7), (8, 8, 4), (8, 8, 5), (8, 8, 6), (8, 8, 7), (8, 8, 8). The *non-square* (*width ≢ height*) NL difficulty levels were assigned in a parallelized fashion (*Set A*) = (5, 4, 4), (4, 5, 5), …, (8, 7, 8), (*Set B*) = (4, 5, 4), (5, 4, 5), …, (7, 8, 8) but still contain all board sizes. Finally, for both sets, one instance of total number of turns (ranging from 4 to 8) was selected randomly. For both the full set A and B, three sets of difficulty levels were created (see Supplementary Material, Appendix): a short version (*width, height, paths, turns*) = (4, 5, 6) × (4, 5, 6) × [4 ≤ *p* ≤ max(width, height)] × [4 ≤ *t* ≤ max(w, h)] = 12 levels, a medium version (*width, height, paths, turns*) = (4, 5, 6, 7) × (4, 5, 6, 7) × [4 ≤ paths ≤ max(width, height)] × [4 ≤ turns ≤ max(width, height)] = 24 levels, and a long version (*width, height, paths, turns*) = (4, 5, 6, 7, 8) × (4, 5, 6, 7, 8) × [4 ≤ paths ≤ max(width, height)] × [4 ≤ turns ≤ max(width, height)] = 40 levels. The reasoning behind creating short, medium, and long versions of NL difficulty levels was based on preliminary experience and mainly due to time considerations and to avoid overburdening participants.

#### Data Preparation, Maze Performance, and Movement Metrics

NL puzzle performance metrics were calculated based on the participants’ completion time, accuracy, and touchscreen interaction (“movement”). For each played maze difficulty level, two data files were stored as text files: (1) time-stamped “screenshots” (two-dimensional arrays) representing every change of state in empty cells from between the initial and solved NL puzzle; and (2) raw touch input data from the tablet-computer consisting of time-stamped *x* and *y* coordinates of touch points.

**Figure 3:**
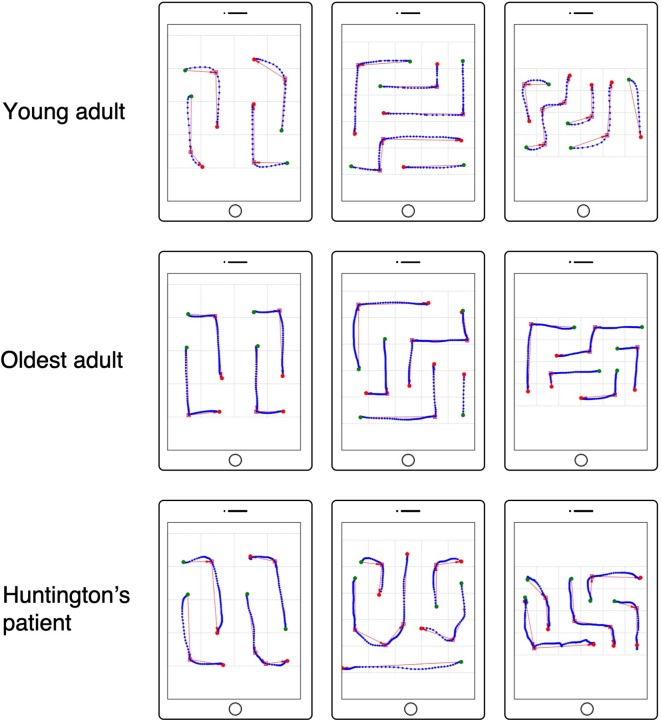
Numberlink motor performance based on tablet touch interaction data. Examples are shown for a young adult (top row), oldest adult (middle row), and patient with Huntington’s disease (HD; bottom row). Touch interaction data are shown for NL puzzle levels solved without errors (false paths) for difficulty levels (width, height, paths, turns) = (4, 4, 4, 4), (5, 5, 5, 5), (6, 4, 5, 6). Green circles indicate the start and red circles indicate the end of a move. Red squares represent turns in the path as detected using the Ramer–Douglas–Peucker algorithm.

First, three response time-based NL performance measures were calculated: total solving time (TST), motor execution time (MET), and mental planning time (MPT). TST was defined as the time elapsed from the initial touch to the solved NL puzzle (see Figure 1). Using the touch input data, single moves (“drag movements”) were defined as a sequence of touch points that fall between a touch down and release event (see Figure 3). For every move, move duration (i.e., the time between touch down and touch release) was calculated (Antal and Szabó, 2016). MET was computed by summing the durations of all moves that were required to complete the NL puzzle. Finally, MPT was calculated by subtracting MET from TST.

Second, two accuracy-based NL performance measures were calculated: the number of false moves and excess moves. False moves were defined as complete or incomplete paths that were not part of the solved maze or, although correct, were deleted before the maze was finally solved. Excess moves were calculated by subtracting the number of paths (i.e., pairs of dots) from the total (i.e., false and correct) number of moves.

Third, movement metrics were computed from the touch data. For this, moves connecting start and end points were extracted. In NL puzzles, moves follow either straight or winding paths. Winding paths change direction from segment to segment depending on the number of turns of the path (see Figures 1, 2). Because movements in NL puzzles are constrained to vertical and horizontal direction, moves for paths with changing directions (i.e., paths involving turns) were segmented into sub-paths based on the detected path turns (see red squares for detected path turns and red arrows for sub-paths in Figure 3) using the Ramer–Douglas–Peucker algorithm (Ramer, 1972; Douglas and Peucker, 1973; Bleier, 2011). For the thus extracted sub-paths and paths without turns (straight), two quantitative movement metrics were calculated: mean velocity (MVE) and movement direction changes (MDC; MacKenzie et al., 2001).

MVE represents the average movement speed. MDC represents the number of direction changes within the axis of the detected sub-paths during drag movements. More recently, these measures have been shown successful in differentiating between groups with and without motor impairment (Keates and Trewin, 2005; Keates et al., 2005; Mertens et al., 2012; Montague et al., 2014; Papatheodorou et al., 2019).

### Procedure

The study consisted of a neuropsychological assessment and a NL puzzle playing session. After written informed consent was obtained, baseline measures of general cognitive ability (MoCA), visual search and visuomotor processing (TMT-A), cognitive flexibility, divided attention, working memory and inhibition (TMT-B), and visuoconstructional ability, planning, and foresight (SMT) were collected.

Next, participants completed a practice block of three NL puzzles: (*w, h, p, t*) = (4, 4, 4, 0), (4, 4, 4, 4), (5, 5, 5, 5) on a tablet-computer (Apple© 12.9” iPad Pro, Apple Inc., Cupertino, CA, United States). Instructions were given that all pairs of same-colored dots must be connected using tap and drag movements to cover all empty cells in the board and that connections will be severed if they intersect. Also, participants were shown the hint button that connects two dots when pressed and encouraged to use a hint should they struggle to solve a puzzle. Thereafter, participants were assigned one of three sets of NL puzzle difficulty levels based on their individual MoCA score and TMT B performance: short version (MoCA < 23, TMT *B* > 120 s), medium version (MoCA ≥ 23, ≤ 28, TMT *B* < 120 s) and long version (MoCA > 28, TMT *B* < 120 s). For each difficulty level, a NL puzzle was selected randomly from the pre-generated NL puzzles. The order of presentation of difficulty levels was randomized in order to avoid learning effects (van Kreveld et al., 2015). After completing the NL puzzle difficulty levels, participants evaluated the usability and their experience with the NL puzzles by filling in the SUS and the Perception of Game Training Questionnaire.

### Statistical Analysis

For group comparison, only the NL difficulty levels from the short version, completed by all participants, were analyzed. Due to non-normally distributed data (visual inspection of histogram and quantile–quantile plots and Shapiro–Wilk tests), statistical differences between the participant groups were performed using the non-parametric Kruskal–Wallis test followed by multiple comparison *post hoc* tests using the pgirmess package (Giraudoux, 2018) in R version 1.1.463 (R Core Team, 2018). As criterion for statistical significance, a probability level of 0.05 was used in *post hoc* comparisons.

To assess the concurrent validity of the NL puzzle task, associations between NL puzzle performance measures and neuropsychological test measures of attentional, visuospatial and visuoconstructional, and executive function and global cognitive ability were tested by correlational analyses (Spearman rank correlation coefficients) using the sjstats package (Lüdecke, 2019). Furthermore, partial correlation analyses (using Spearman rank correlation coefficients), controlling for participant age, were performed using the ppcor package (Kim, 2015).

Difficulty level manipulation was examined using correlational analyses (Spearman rank correlation coefficients) between total time to solve the puzzle and difficulty level. Difficulty levels were ordered based on the set size (width × height) and the number of paths of the NL puzzles. Only data from the young and older adults who completed the full range of manipulated difficulty levels were analyzed using separate correlations for young and older adults.

## Results

### Results for Demographic Variables and Neuropsychological Tests

Participant demographic variables and neuropsychological test data are shown in Table 1. Groups differed significantly in age ($\chi_{\left(4\right)}^{2}$ = 568.21; *p* < 0.001), but not in years of education (*F*_(4, 38)_ = 1.744, *p* = 0.161). Neuropsychological test measures revealed significant group differences in the TMT-A completion time ($\chi_{\left(4\right)}^{2}$ = 29.43; *p* < 0.001) and errors ($\chi_{\left(4\right)}^{2}$ = 11.26; *p* = 0.024), the TMT-B completion time ($\chi_{\left(4\right)}^{2}$ = 32.13; *p* < 0.001) and errors ($\chi_{\left(4\right)}^{2}$ = 13.04; *p* = 0.01) and number correct ($\chi_{\left(4\right)}^{2}$ = 8.62; *p* = 0.07), the SMT solving time ($\chi_{\left(4\right)}^{2}$ = 32.56; *p* < 0.001) and errors ($\chi_{\left(4\right)}^{2}$ = 13.52; *p* < 0.01) and the MoCA score ($\chi_{\left(4\right)}^{2}$ = 19.18; *p* < 0.001). *Post hoc* analyses (multiple comparison tests, *p* < 0.05) that young adults were significantly faster on the TMT-A and TMT-B than oldest adults (TMT-A *p* < 0.001, TMT-B *p* < 0.001) and patients with HD (TMT-A *p* = 0.021, TMT-B *p* = 0.019). Furthermore, young adults were significantly faster on the TMT-B than old adults (*p* = 0.001). SMT solving time was significantly different between young adults and older adults (*p* < 0.001), oldest adults and patients with HD (*p* = 0.009).

### Results for Perception and Usability of the NL Puzzle Game

Ratings of enjoyment ($\chi_{\left(4\right)}^{2}$ = 3.81; *p* = 0.43), challenge ($\chi_{\left(4\right)}^{2}$ = 8.75; *p* = 0.07), frustration ($\chi_{\left(4\right)}^{2}$ = 8.98; *p* = 0.06), and motivation ($\chi_{\left(4\right)}^{2}$ = 2.36; *p* = 0.67) for the NL puzzle game did not differ significantly between groups. There was a significant difference in usability ratings between groups ($\chi_{\left(4\right)}^{2}$ = 12.04; *p* = 0.02), but *post hoc* comparisons failed to show any significant differences between groups (YA vs. OA, OOA vs. PD, OOA vs. HD, PD vs. HD: *p* = 1.000, YA vs. OOA *p* = 0.140, YA vs. PD *p* = 0.609, YA vs. HD *p* = 0.355, OA vs. OOA *p* = 0.228, OA vs. PD *p* = 0.689, OA vs. HD *p* = 0.414). Overall, individual SUS ratings ranged from 67.50 (“good”) to 100.00 (“best”) with a mean SUS rating of 86.43 (“excellent”; Bangor et al., 2008).

### Results for NL Performance Measures

**Figure 4:**
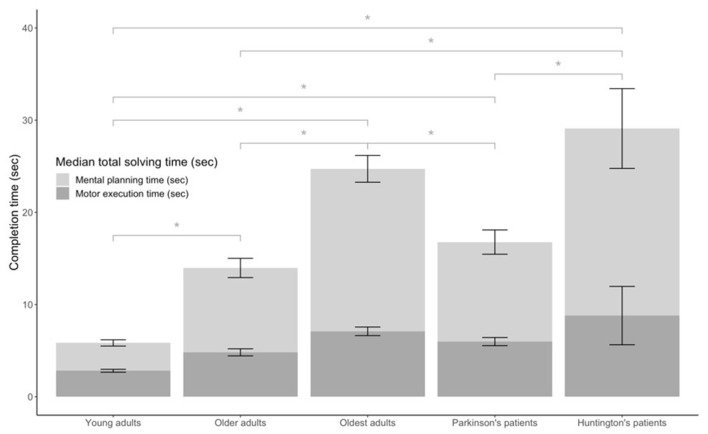
Median solving times of Numberlink puzzles for young, older, and oldest adults, and patients with Parkinson’s and Huntington’s disease. Total solving time (TST) is shown separately as the time needed to connect paths motor execution time (MET) and time where no movements were made mental planning time (MPT), *significant difference at the 0.05 level.

NL puzzle game performance measures by group can be found in Table 2 and are displayed in Figure 4. For the time-based NL performance measures, there were significant group differences in TST ($\chi_{\left(4\right)}^{2}$ = 419.51, *p* < 0.001), MET ($\chi_{\left(4\right)}^{2}$ = 324.72, *p* < 0.001), and MPT ($\chi_{\left(4\right)}^{2}$ = 410.97, *p* < 0.001). *Post hoc* analyses showed that both total solving (TST: YA vs. OA, YA vs. OOA, YA vs. PD, YA vs. HD, OA vs. OOA *p* < 0.001, OOA vs. PD *p* = 0.034, PD vs. HD: *p* = 0.002) and MPTs (MPT: YA vs. OA, YA vs. OOA, YA vs. HD, *p* < 1.000, YA vs. PD *p* = 0.009, OA vs. OOA *p* = 0.001, OOA vs. PD *p* = 0.015, OA vs. HD = 0.034, PD vs. HD: *p* = 0.008) were significantly different between all groups (multiple comparison test, *p* < 0.05), except between older adults and patients with PD (TST *p* = 1.000, MPT *p* = 1.000) and between oldest adults and patients with HD (TST *p* = 1.000, MPT *p* = 1.000). MET (MET: YA vs. OA, YA vs. OOA, YA vs. HD, *p* < 0.001, YA vs. PD *p* = 0.002, OA vs. OOA *p* = 0.003, OA vs. HD = 0.008, PD vs. HD: *p* = 0.043) differed significantly between all groups (*p* < 0.05) with the exception of older adults and Parkinson’s patients, oldest adults and Parkinson’s patients, and oldest adults and Huntington’s patients that were not significantly different.

**Table 2:** Time, accuracy, and movement-based performance measures for the Numberlink puzzle game by group.

	YA (*n* = 18)	OA (*n* = 14)	OOA (*n* = 14)	PD (*n* = 4)	HD (*n* = 5)	Statistics
	*Numberlink puzzle time-based performance measure*					
TST (s)	7.55 (7.34)	20.03 (17.97)	33.88 (20.68)	19.61 (11.81)	52.75 (57.49)	*χ*^2^ = 419.15^Z^, *p* < 0.001^Z,A,B,C,D,E^, 0.034^H^, 0.002^J^, 1.000^F,I^
MET (s)	3.43 (2.45)	5.91 (5.21)	9.32 (5.56)	6.52 (3.21)	19.25 (25.50)	$\chi_{\left(4\right)}^{2}$ = 324.72, *p* < 0.001^Z,A,B,D^, 0.002^C^, 0.003^E^, 0.008^G^, 0.043^J^, 1.000^F,H,I^
MPT (s)	4.12 (5.25)	14.12 (14.19)	24.56 (17.37)	13.09 (9.58)	33.50 (34.88)	$\chi_{\left(4\right)}^{2}$ = 410.97, *p* < 0.001^Z,A,B,D^, 0.009^C^, 0.001^E^, 0.015^H^, 0.034^G^, 0.008^J^, 1.000^F,I^
	*Numberlink puzzle accuracy-based performance measures*					
False moves	0.85 (3.02)	1.12 (3.58)	2.01 (3.65)	1.25 (2.24)	6.49 (13.62)	$\chi_{\left(4\right)}^{2}$ = 50.03, *p* < 0 001^Z^, 0.001^B^, 0.005^D^, 0.017^E^, 0.068^G^, 1.000^A,C,F,H,I,J^
Excess moves	0.90 (3.14)	1.32 (4.26)	2.13 (3.91)	1.42 (2.80)	7.31 (15.53)	$\chi_{\left(4\right)}^{2}$ = 42.44, *p* < 0.001^Z^, 0.001^B^, 0.017^D^, 0.039^E^, 1.000^A,C,F,G,H,I,J^
Number of hints	0.00 (0.00)	0.00 (0.00)	0.00 (0.00)	0.00 (0.00)	0.08 (0.62)	$\chi_{\left(4\right)}^{2}$ = 2.24, *p* = 0.69^Z^
	*Numberlink puzzle movement-based performance measures*					
MVE (pixels/s)	2658.49 (1008.30)	1701.04 (916.75)	1150.91 (661.12)	1664.62 (861.92)	1064.91 (614.01)	$\chi_{\left(4\right)}^{2}$ = 289.68, *p* < 0.001^Z,A,B,D^, 0.001^C^, 0.029^E^, 0.008^G^, 0.071^J^, 0.082^H^, 1.000^F,I^
MDC	98.79 (189.82)	155.83 (208.54)	196.07 (135.92)	189.48 (113.15)	540.67 (911.05)	$\chi_{\left(4\right)}^{2}$ = 234.94, *p* < 0.001^Z,B^, 0.010^A^, 0.028^C^, 0.001^D^, 0.044^E^, 0.013^G^, 1.000^F,H,I,J^

*All values, mean (SD), *p* value < 0.05 is significant. YA, Young Adults; OA, Older Adults; OOA, Old oldest Adults; PD, Parkinson’s Disease; HD, Huntington’s disease; TST, total solving time; MET, motor execution time; MPT, mental planning time; MVE, mean velocity; MDC, mean number of movement direction changes; ^Z^comparison across all groups df = 4, ^A^YA vs. OA, ^B^YA vs. OOA, ^C^YA vs. PD, ^D^YA vs. HD, ^E^OA vs. OOA, ^F^OA vs. PD, ^G^OA vs. HD, ^H^OOA vs. PD, ^I^OOA vs. HD, ^J^PD vs. HD*.

Results for accuracy-based NL performance indicators further suggest significant group differences in the number of false ($\chi_{\left(4\right)}^{2}$ = 50.03, *p* < 0.001) and excess moves ($\chi_{\left(4\right)}^{2}$ = 42.44, *p* < 0.001) moves. There were no significant group differences in the number of used hints ($\chi_{\left(4\right)}^{2}$ = 2.24, *p* = 0.69). The number of false moves were significantly different between the young and oldest adults (*p* = 0.001), young adults and patients with HD (*p* = 0.005), as well as between the older adults and the oldest adults (*p* = 0.017) and the older adults and patients with HD. *Post hoc* comparisons further revealed that patients with HD made significantly more excess moves than both young (*p* = 0.017) and older adults. Oldest adults further made significantly more excess moves than young adults (*p* = 0.001).

### Results for NL Puzzle Movement Metrics

Analysis of movement-based measures when playing the NL puzzles, revealed significant differences between groups in average movement velocity ($\chi_{\left(4\right)}^{2}$ = 289.68, *p* < 0.001) and average number of MDC ($\chi_{\left(4\right)}^{2}$ = 234.94, *p* < 0.001). *Post hoc* group comparisons revealed significant differences in average movement velocity (MVE: YA vs. OA, YA vs. OOA, YA vs. HD, *p* < 0.001, YA vs. PD *p* = 0.001, OA vs. OOA *p* = 0.029, OA vs. HD *p* = 0.008) between all groups (*p* < 0.05) except with older adults and patients with PD, and oldest adults compared to patients with HD and PD. In terms of the number of MDC, all group comparisons (MDC: YA vs. OA *p* = 0.010, YA vs. OOA *p* < 0.001, YA vs. PD *p* = 0.028, YA vs. HD *p* = 0.001, OA vs. OOA *p* = 0.044, OA vs. HD: *p* = 0.013) were significant, except for patients with HD compared to oldest adults and patients with PD and patients with PD compared to older and oldest adults.

### Correlation Between NL Puzzle Performance and Neuropsychological Test Measures

Correlational analyses (see Table 3) revealed significant associations between time-based NL puzzle performance (median TST) and performance in visuomotor and visuospatial (TMT-A time), executive (shifting, inhibition; TMT-B time), visuoconstructional and executive (planning and foresight) function (SMT time), and global cognitive ability (MoCA). Partial correlations controlling for age remained significant. Similarly, accuracy-based NL puzzle measures (number of false and excess moves) were significantly correlated with TMT-A and -B time, SMT time, and the MoCA score. However, when controlling for age with partial correlation, the correlations with SMT time and MoCA did not remain significant. Finally, movement-based measures from the NL puzzles (MVE, MDC) all showed significant correlations with SMT time, TMT-A and -B time, and MoCA score. After controlling for age, these correlations remained significant with the exception of correlations between MDC and global cognitive ability (MoCA).

**Table 3:** Correlations between Numberlink puzzle performance and movement metrics and neuropsychological test measures.

	SMT time (s)		TMT A time (s)		TMT B time (s)		MoCA score	
Variables	Simple Controlled *ρ* (*p* value)	Correlation for age *ρ* (*p* value)	Simple Controlled *ρ* (*p* value)	Correlation for age *ρ* (*p* value)	Simple Controlled *ρ* (*p* value)	Correlation for age *ρ* (*p* value)	Simple Controlled *ρ* (*p* value)	Correlation for age *ρ* (*p* value)
TST	0.813***	0.58***	0.781***	0.596***	0.793***	0.633***	−0.637***	−0.426**
False moves	0.447**	0.23^ns^	0.485***	0.316*	0.479**	0.338*	−0.342*	−0.158^ns^
Excess moves	0.441	0.22^ns^	0.486***	0.316*	0.482**	0.336*	−0.335*	−0.149^ns^
MVE	−0.787***	−0.61***	−0.802***	−0.609***	−0.746***	−0.598***	0.599***	0.398***
MDC	0.684***	0.459**	0.691***	0.509***	0.711***	0.575***	−0.497***	−0.27^ns^

SMT, Snellgrove maze task; TMT A, Trail making test part A; TMT B, Trail making test part B; MoCA, Montreal cognitive assessment; TST, total solving time; MVE, Mean velocity; MDC, mean number of movement direction changes; ns, not significant; ***significant at the 0.001 level, **significant at the 0.01 level, *significant at the 0.05 level.

### Results for Task Difficulty Manipulation

**Figure 5:**
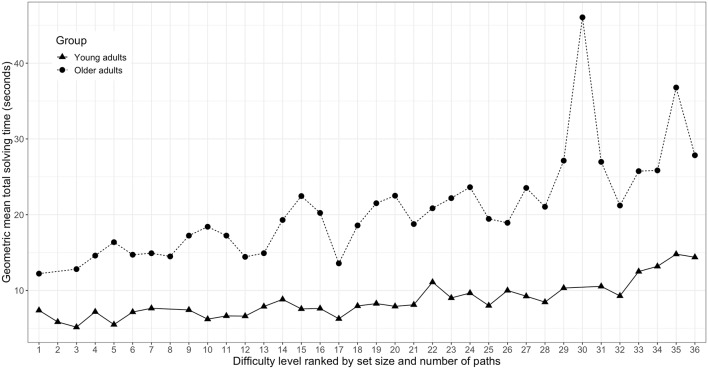
Total Numberlink puzzle solving time as a function of ranked difficulty level for young (*n* = 18) and older adults (*n* = 14). Difficulty levels are defined by set size and the number of paths of the respective Numberlink puzzles.

Total NL puzzle solving time as a function of difficulty level are shown separately for young and older adults that completed the full range of NL difficulty levels in Figure 5. Separate Spearman correlation analyses between total puzzle solving time and ranked difficulty levels revealed a significant positive association for both the young adults (*r*_(17)_ = 0.583, *p* < 0.001) and the older adults (*r*_(13)_ = 0.496, *p* < 0.001).

## Discussion

The aim of this study was to evaluate the feasibility and preliminary validity of a maze-like NL puzzle video game as a tool to assess cognitive and motor differences in older adults and patients with NDDs. Results from this study show that NL puzzles are enjoyable, motivating, and user-friendly for older adults and patients with motor difficulties in neurodegenerative diseases. Analyses of game-based measures of cognitive and motor performance showed significant differences in executive and motor function within the different age groups and between the disease groups. However, there were no differences in total solving, mental planning, and MET between HD and the oldest age (OOA) group and between PD and the older age (OA) group. Initial concurrent validation showed that the NL puzzle game performance correlates with a standardized maze task (SMT) and tests for cognitive abilities relevant to cognitive decline due to aging and NDD. This study further shows that characteristics of the game can be adjusted to create graded levels of difficulty.

Analyses of performance on the played NL puzzle game levels showed significant age-group differences. Overall, older adults took longer to solve NL puzzles than young adults, and oldest adults were slower compared to older adults. The differences in NL puzzle performance between the age groups are consistent with age-related changes in motor and executive functioning (Zhang et al., 2007). One explanation for the effect of age on NL puzzle game performance might simply be a decline in motor processing speed with age (Salthouse, 2000; Ebaid et al., 2017). Findings from this study do show that MET (i.e., total time needed to manually connect paths in the maze) and average velocity of these movements in the NL puzzle game were significantly different between young, older, and oldest adults. However, the movement velocity and MDC did not differ between the oldest groups and the neurodegenerative disease groups. Reasons may lie in the visuoconstructional (Snellgrove, 2005) and perceptual-motor function (Zhao and Marquez, 2013) skills required for NL puzzle games. The perceptual-motor skills decline with age and neurodegenerative diseases. For PD and HD patients, the UPDRS-Motor score quantifies the decline in motor skills. It would be advisable to quantify the manual dexterity of older and oldest adults so that they can be adjusted for their game-based motor performances. PD patients recruited in our study were in their early disease stage and had very low UPDRS-Motor scores, which is seen clearly in their motor performances.

However, our finding of age-group differences in MPT (i.e., subtracting the MET from the TST) replicates previous studies that showed age effects on maze solving ability (Koss et al., 1991; Krishnasamy and Unsworth, 2011; de Souza et al., 2013), even when controlling for motor processing speed. The age effect on maze solving ability has been suggested to reflect age-related difficulties in executive and visuoconstructive functioning (Krishnasamy and Unsworth, 2011; de Souza et al., 2013). Interestingly, studies have suggested that executive deficits with aging are not general, but specifically affect planning ability that is usually measured using maze tasks (Zhang et al., 2007; Brandt et al., 2009) and strongly associated with everyday functioning in older age (Lewis and Miller, 2007). Furthermore, solving the NL puzzle game requires sequencing of motor actions to organize and sequence multiple paths that need to be connected in order to solve NL puzzles (Laurentiz, 2018). Similar to planning ability, sequencing motor actions is increasingly dependent on executive function and declines with age (Niermeyer et al., 2017).

Compared to young and older adults, patients with HD were significantly slower in both total solving, mental planning, and MET. This finding is underscored by the fact that HD patients made significantly more errors and excess moves when solving NL puzzles. Taken together, the difficulties of HD patients with solving maze-like NL puzzles are consistent with previous findings and reflect declines in psychomotor, visuospatial and executive function that occur throughout HD (Fedio et al., 1979; Montoya et al., 2006). Contrary to a previous study (Mimura et al., 2006), this study found no difference between PD patients and older adults in total solving, mental planning, and MET. This suggests that the PD patients in this study suffered no deficits in psychomotor and planning aspects of executive function. This finding was mirrored in the assessments done using the standardized SMT. PD patients recruited in our study had a good cognitive status, as indicated in their MoCA score (26.25 ± 3.40). Their MDS-UPDRS-III scores (17.75 ± 5.38) were significantly lower than the HD patients and displayed very few motor deficits. This was reflected in the game performance results such as mental planning and motor execution. However, PD medications can affect both their cognitive and motor skill levels, which cannot be ruled out.

As the MPT is TST minus the execution time, we could rule out a confounding effect of motor performance (Ebaid et al., 2017). In addition, we found identical group differences for game-based motor performance measures (MET and velocity and direction changes of movements), only this time, Parkinson’s patients were not different from oldest adults. Interestingly, these findings parallel earlier studies that assessed touch interactions on mobile devices in older adults and Parkinson’s patients (Keates and Trewin, 2005; Montague et al., 2014) and are supported by a recent viewpoint paper claiming that game-based movement measures reflect psychomotor deficits in age-related neurodegenerative diseases such as AD, PD, and HD (Mandryk and Birk, 2019).

The concurrent validity findings in this study show that game performance measures from the NL puzzle game were significantly correlated with performance on tests for visuomotor, visuospatial, executive, and visuoconstructional function. Game performance was also associated with global cognitive ability. These findings confirm recent suggestions that video games incorporate elements and cognitive challenges shared with psychological tasks (Holmgard et al., 2016). Furthermore, results indicate that game-based measures of cognitive and motor performance can be used to assess and monitor cognitive function in normal aging and neurodegenerative diseases (Koo and Vizer, 2019; Mandryk and Birk, 2019). Significant correlations between NL-based time and movement performance with tests of psychomotor, attentional, visuoconstructional, and executive functions support our assumption that NL puzzles share cognitive components with classical maze tasks (Snellgrove, 2005; Kirsch et al., 2006). Thus, a digital maze test like the NL can be used as a nonverbal intelligence test to estimate planning and inhibition. Moreover, they can overcome inconsistencies in interrater reliability seen in standard paper-based maze tests and can be deployed in a familiar user environment. The continuous and automatic data recording with digital technology will allow easy data collection of errors, corrections, and attempts of each user.

Our results on the usability and perception of the NL puzzles showed that they are enjoyable, motivating, user-friendly, and easy to use for both the healthy age groups and the neurodegenerative groups. However, there was a slight trend in rating the game session as challenging and frustrating, which is driven by perception ratings of the PD and HD patients. On the one hand, this might simply reflects the disease-related motor challenges in performance. On the other hand, it might be that it was harder for PD and HD patients to adapt to the difficulty levels. Adapting the difficulty levels to a person’s performance might resolve a part of this problem and also ensure that the puzzle games are equally challenging for players with different levels of cognitive ability (Lankoski, 2015; Holmgard et al., 2016).

Some limitations of this study need to be addressed: first, the sample size of this study is small, particularly for the groups with neurodegenerative disorders. Moreover, the level of progression of disease within the different neurodegenerative disease was not controlled for in this study. Therefore, validation studies with larger sample size are definitely needed to further confirm these initial findings. Second, our findings are based on comparing the performance on a limited set of NL puzzle difficulty levels. A possible solution to this would be to use computerized adaptive cognitive testing that adapts the difficulty of the task to the individual’s level of cognitive ability by selecting test items from graded difficulty levels. Using the pre-generated NL puzzle difficulty levels from this study, this would allow to better detect subtle changes in executive and motor function across a wider range of maze-like NL puzzle difficulties and avoid both floor and ceiling effects. Third, this study is cross-sectional, and participants play the NL only once and for the first time.

To sum up, this study supports recent suggestions that game-based data from playing a commercial maze-like puzzle game provides potential “digital biomarkers” to assess cognitive function and cognitive decline over time. In particular, NL puzzle games seem promising for capturing changes in visuomotor, visuoconstructional, and executive function related to aging and neurodegeneration.

## Data Availability Statement

Data pertaining to the obtained results may be provided upon request.

## Ethics Statement

The studies involving human participants were reviewed and approved by The ethics committee of Canton Bern and Ethics Committee of Northwest and Central Switzerland. The patients/participants provided their written informed consent to participate in this study.

## Author Contributions

TN, RM, and PU contributed to the conception and organization of the research. AC, TN, TV, JM, J-MB, and PU participated in the execution and data collection. AC, NS, AB, and PU designed the data analysis and statistical methods. AC wrote the first draft of the manuscript. All authors participated in the review and critical review of the manuscript.

## Conflict of Interest

The authors declare that the research was conducted in the absence of any commercial or financial relationships that could be construed as a potential conflict of interest.
