# Supplementary material for: Development and Evaluation of Maze-Like Puzzle Games to Assess Cognitive and Motor Function in Aging and Neurodegenerative Diseases
Source: Front Aging Neurosci. 2020 Apr 21;12:87. doi: 10.3389/fnagi.2020.00087 (PMC7188385; doi:10.3389/fnagi.2020.00087)
Supplement: Supplementary file 1 [file Table_1.DOCX]

|  | **Numberlink A** | | | | **Numberlink B** | | | |  |
| --- | --- | --- | --- | --- | --- | --- | --- | --- | --- |
| Level | Width | Height | Set size | Paths | Width | Height | Set size | Paths | Version |
| 1 | 4 | 4 | 16 | 4 | 4 | 4 | 16 | 4 | Short Version  (12 difficulty levels) |
| 2 | 5 | 4 | 20 | 4 | 4 | 5 | 20 | 4 |  |
| 3 | 4 | 5 | 20 | 5 | 5 | 4 | 20 | 5 |  |
| 4 | 5 | 5 | 25 | 4 | 5 | 5 | 25 | 4 |  |
| 5 | 5 | 5 | 25 | 5 | 5 | 5 | 25 | 5 |  |
| 6 | 6 | 4 | 24 | 4 | 4 | 6 | 24 | 4 |  |
| 7 | 4 | 6 | 24 | 5 | 6 | 4 | 24 | 5 |  |
| 8 | 6 | 4 | 24 | 6 | 4 | 6 | 24 | 6 |  |
| 9 | 6 | 5 | 30 | 5 | 5 | 6 | 30 | 5 |  |
| 10 | 5 | 6 | 30 | 6 | 6 | 5 | 30 | 6 |  |
| 11 | 6 | 6 | 36 | 5 | 6 | 6 | 36 | 5 |  |
| 12 | 6 | 6 | 36 | 6 | 6 | 6 | 36 | 6 |  |
| 13 | 7 | 4 | 28 | 4 | 4 | 7 | 28 | 4 | Medium Version  (24 difficulty levels) |
| 14 | 4 | 7 | 28 | 5 | 7 | 4 | 28 | 5 |  |
| 15 | 7 | 4 | 28 | 6 | 4 | 7 | 28 | 6 |  |
| 16 | 4 | 7 | 28 | 7 | 7 | 4 | 28 | 7 |  |
| 17 | 5 | 7 | 35 | 5 | 7 | 5 | 35 | 5 |  |
| 18 | 7 | 5 | 35 | 6 | 5 | 7 | 35 | 6 |  |
| 19 | 5 | 7 | 35 | 7 | 7 | 5 | 35 | 7 |  |
| 20 | 6 | 7 | 42 | 5 | 7 | 6 | 42 | 5 |  |
| 21 | 7 | 6 | 42 | 6 | 6 | 7 | 42 | 6 |  |
| 22 | 6 | 7 | 42 | 7 | 7 | 6 | 42 | 7 |  |
| 23 | 7 | 7 | 49 | 6 | 7 | 7 | 49 | 6 |  |
| 24 | 7 | 7 | 49 | 7 | 7 | 7 | 49 | 7 |  |
| 25 | 4 | 8 | 32 | 5 | 8 | 4 | 32 | 5 | Long Version  (40 difficulty levels) |
| 26 | 8 | 4 | 32 | 6 | 4 | 8 | 32 | 6 |  |
| 27 | 4 | 8 | 32 | 7 | 8 | 4 | 32 | 7 |  |
| 28 | 8 | 4 | 32 | 8 | 4 | 8 | 32 | 8 |  |
| 29 | 5 | 8 | 40 | 5 | 8 | 5 | 40 | 5 |  |
| 30 | 8 | 5 | 40 | 6 | 5 | 8 | 40 | 6 |  |
| 31 | 5 | 8 | 40 | 7 | 8 | 5 | 40 | 7 |  |
| 32 | 8 | 5 | 40 | 8 | 5 | 8 | 40 | 8 |  |
| 33 | 8 | 6 | 48 | 6 | 6 | 8 | 48 | 6 |  |
| 34 | 6 | 8 | 48 | 7 | 8 | 6 | 48 | 7 |  |
| 35 | 8 | 6 | 48 | 8 | 6 | 8 | 48 | 8 |  |
| 36 | 8 | 7 | 56 | 6 | 7 | 8 | 56 | 6 |  |
| 37 | 8 | 7 | 56 | 7 | 7 | 8 | 56 | 7 |  |
| 38 | 8 | 7 | 56 | 8 | 7 | 8 | 56 | 8 |  |
| 39 | 8 | 8 | 64 | 7 | 8 | 8 | 64 | 7 |  |
| 40 | 8 | 8 | 64 | 8 | 8 | 8 | 64 | 8 |  |

Appendix

Difficulty levels generated for the Numberlinks sets A and B: short version 12 levels, medium version 24 levels, long version 40 levels.
